# Exploiting Complexity Information for Brain Activation Detection

**DOI:** 10.1371/journal.pone.0152418

**Published:** 2016-04-05

**Authors:** Yan Zhang, Jiali Liang, Qiang Lin, Zhenghui Hu

**Affiliations:** 1 College of Optical and Electronic Technology, China Jiliang University, Hangzhou 310018, China; 2 Center for Optics and Optoelectronics Research, College of Science, Zhejiang University of Technology, Hangzhou 310023, China; Xuanwu Hospital, Capital Medical Universty, CHINA

## Abstract

We present a complexity-based approach for the analysis of fMRI time series, in which sample entropy (SampEn) is introduced as a quantification of the voxel complexity. Under this hypothesis the voxel complexity could be modulated in pertinent cognitive tasks, and it changes through experimental paradigms. We calculate the complexity of sequential fMRI data for each voxel in two distinct experimental paradigms and use a nonparametric statistical strategy, the Wilcoxon signed rank test, to evaluate the difference in complexity between them. The results are compared with the well known general linear model based Statistical Parametric Mapping package (SPM12), where a decided difference has been observed. This is because SampEn method detects brain complexity changes in two experiments of different conditions and the data-driven method SampEn evaluates just the complexity of specific sequential fMRI data. Also, the larger and smaller SampEn values correspond to different meanings, and the neutral-blank design produces higher predictability than threat-neutral. Complexity information can be considered as a complementary method to the existing fMRI analysis strategies, and it may help improving the understanding of human brain functions from a different perspective.

## Introduction

fMRI refers to the MRI-based detection of hemodynamic changes associated with neural activity. The goal of fMRI data analysis is to search for relevant information of the neural activation sites as well as their relationship, which are induced by the experiment. Generally, fMRI activation detection techniques can be classified as model-driven approaches that perform statistical validation of prior hypotheses, and data-driven methods that mainly extract temporally/spatially features in the data, such as decorrelation [1], independence [2] and similarity [3].

Nevertheless, while one surveys fMRI signals, a detail must not be ignored that these signals, like many other physiological time series, commonly exhibit extremely inhomogeneous and non-stationary fluctuations in an irregular and complex manner [4, 5]. We hypothesize that these complexity/regularity could be modulated in pertinent cognitive tasks, and they may change through experimental paradigms. Thus, direct assessment of the fMRI signal complexity and regularity may offer certain physiological insights for brain research [6].

The historical development of mathematics to quantify complexity and regularity has centered around various types of entropy measures [7–10]. Entropy in this context is defined as a measure of uncertainty of information in a statistical description of a system, with greater entropy often associated with more randomness and less system order. In general, a huge number of data would be demanded to achieve convergence in entropy algorithms. Approximate entropy (ApEn) and sample entropy (SampEn) belong to a recently developed family of parameters and statistics for quantifying system complexity and regularity, and possess the significant property that they can be applied to a relatively small amount of serial data, even no more than 72 points [11–13], to assess system regularity and to distinguish abnormal from normal data, where classical moment statistics approaches fail to show meaningful differences. Since most fMRI data are recorded for 100 or so temporal volumes, ApEn and especially SampEn are thus especially attractive for fMRI time series complexity assessment [14, 15]. Particularly, linear correlation analysis of BOLD signal in time/frequency domain dissects underlying functional connectivity activity but is very sensitive to missing data points [16, 17]. However SmapEn little affected by loss of the data, the practical limit that we might encounter [18]. This robust feature has the potential to accommodate unpredictable errors in the image acquisition or transmission (e.g. loss of the data). The decrease of entropy represents reduced variability in BOLD signal that might reflect dysfunction of autonomic nervous system. Sampan may find use as a general estimate of the health of the brain. Regardless of the underlying mechanism, it is important in clinical medicine, as SampEn can be considered a candidate measure for evaluating brain functions.

In this paper, SampEn, which provides a mathematical quantification of regularity, was applied to voxel-based analysis of fMRI sequences from two block design dataset. Subsequently, a non-parametric statistical strategy was used to draw inference related to different paradigms. The statistical results of complexity and regularity of the population were compared with a typical GLM method, SPM12(http://www.fil.ion.ucl.ac.uk/spm) [19, 20]. Our methodology may give more comprehensive description of the dynamical process embedded in the fMRI data, and should be a valuable complementary approach to the existing analysis methods.

## Methodology

### Experiment Setup and Data Preprocessing

#### Subjects and Image Acquisition

It is worth mentioning that the fMRI experiment was performed with the approval of the Health Science Research Ethics Committee of China Jiliang University, and the participants provided written informed consent before beginning the experiment. Nine healthy subjects (seven male, mean age 32) were included in this study. Participants underwent scanning while listening passively to (i): emotionally neutral word alternating with no word as the control condition (neutral-blank), and (ii): threat-related words alternating with emotionally neutral word as the experimental condition (threat-neutral). Each word was presented in pseudorandom order in 16s blocks of 12 words of the same type. Eight alternating blocks of neutral words were presented for about 256s.

Computer produced stimuli were presented through sound attenuating earphones to the subject who had his eyes closed. Participant was instructed on the tasks prior to scanning and was provided with a brief practice period.

Functional images were acquired on a 1.5-Tesla scanner (Marconi EDGE ECLIPSE) using a standard fMRI gradient echo echo-planar imaging (EPI) protocol (TE, 40ms; TR, 2500ms; flip angle, 90°; NEX, 1; FOV, 24cm; resolution, 64 × 64 matrix). Sixteen contiguous 6-mm-thick, 0.5-mm-intervals were acquired to provide a coverage of the entire brain. Scanning was synchronized with the onset of the first stimulate so that 8 images were acquired during each 16s trail with a total of 128 images per run (8 trails per run).

An additional baseline block (16 images, 32s) was added to the beginning of the run to allow the MR signal to reach equilibrium and familiar scan noise for the subject, and was discarded from further analysis.

#### Data Preprocessing

SPM12 was used for the fMRI data preprocessing [21]. Each image volume was realigned to the first volume. The resultant image volumes were spatially smoothed with a 6-mm FWHM Gaussian kernel to decrease spatial noise, and spatially normalized into the standard MNI atlas space.

### Measurement of Complexity: ApEn and SampEn

ApEn can be used to measure the complexity and regularity of time series dynamics [11, 22]. It is a biased statistic due to counting self-matches to avoid the occurrence of *ln*(0), that is, the expected value of ApEn(*m*, *r*, *N*) is asymptotically equal to the parameter, which is estimated as the number of data *N* increases [23, 24].

This implies that a certain number of data points is needed to achieve reasonably precise estimates. For some biological signals such as fMRI, however, this requirement is difficult to satisfy since typically only 100 or so imaging volumes are recorded. To rid of this bias, sample entropy was introduced as a modification of the ApEn [12]. Considering time series {*x*(1), *x*(2), …, *x*(*N*)}, for the embedding dimensionality *m*, the embedding vector *u*(*i*) in the reconstructed phase space $R^{m}$ is *u*(*i*) = [*x*(*i*), *x*(*i* + 1), ⋯, *x*(*i* + *m* − 1)]. Next, define for each *i*, 1 ≤ *i* ≤ *N* − *m*,

$$\begin{array}{c} A_{i}^{m}\left(r\right)=\frac{1}{N-m-1}\sum_{\overset{j=1}{j\neq i}}^{N-m}{\Theta (r-||u\left(i\right)-u\left(j\right)||}_{1}) \end{array}$$ (1)

where Θ is the discontinuous step Heaviside function

$$\begin{array}{c} \Theta \left(x\right)=\left\{\begin{array}{cc} 0, & \text{for}\;x<0\\ 1, & \text{for}\;x\geq 0 \end{array}\right. \end{array}$$ (2)

Here, *r* specifies a tolerance which is usually expressed as a fraction of the standard deviation (SD) of the data set, and ||.||_1_ is the maximum absolute column sum of matrix norms [25]:

$$\begin{array}{c} {\left||u|\right|}_{1}=\underset{j}{max}\sum_{i=1}^{p}\left|u_{ij}\right|\text{,}\;\text{for}\;p\times q\;\text{matrix.} \end{array}$$ (3)

In particular, ${\left||u\left(i\right)|\right|}_{1}=\underset{j}{max}\left|x\left(j\right)\right|$ for row vector, *u*(*i*) = [*x*(*i*), *x*(*i* + 1), ⋯, *x*(*i* + *m* − 1)]. Similarly, in the reconstructed phase space $R^{m+1}$, we define

$$\begin{array}{c} B_{i}^{m}\left(r\right)=\frac{1}{N-m-1}\sum_{\overset{j=1}{j\neq i}}^{N-m}{\Theta (r-||u\left(i\right)-u\left(j\right)||}_{1}) \end{array}$$ (4)

It is worthwhile to note that $A_{i}^{m}\left(r\right)$ and $B_{i}^{m}\left(r\right)$ have exactly the same form of definition, but are defined in different space. With the definitions of

$$\begin{array}{c} A^{m}\left(r\right)=\frac{1}{N-m}\sum_{i=1}^{N-m}A_{i}^{m}\left(r\right),\;B^{m}\left(r\right)=\frac{1}{N-m}\sum_{i=1}^{N-m}B_{i}^{m}\left(r\right) \end{array}$$ (5)

we can now define the sample entropy as

$$\begin{array}{c} \text{SampEn}\left(m,r,N\right)=ln[A^{m}\left(r\right)/B^{m}\left(r\right)] \end{array}$$ (6)

**Fig 1:**
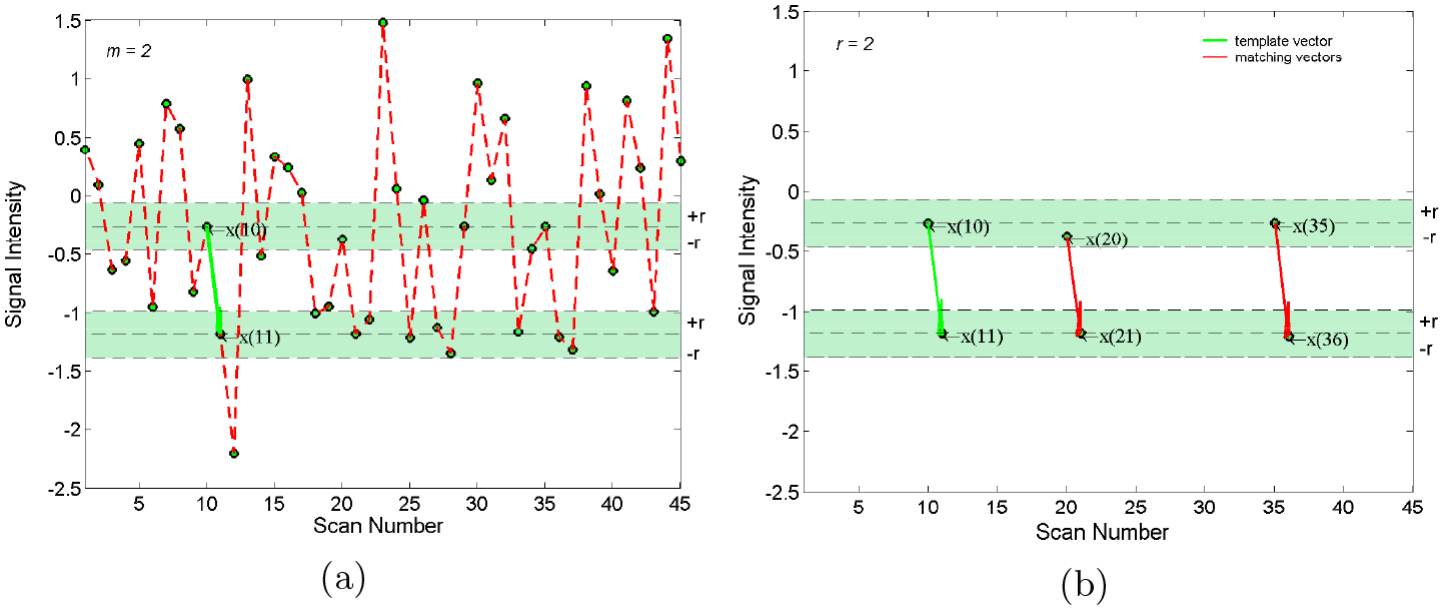
Illustration of SampEn algorithm with the embedding dimension *m* = 2. The colored bands show the tolerance regions *r*. (a) Green arrow denotes template vector *u*(10) = [*x*(10), *x*(11)]. (b) Only vectors *u*(20) = [*x*(20), *x*(21)], *u*(35) = [*x*(35), *x*(36)] (red arrow) falling into these bands were counted to match the template vector: *u*(10) = [*x*(10), *x*(11)].

The basic idea of SampEn algorithm is illustrated with a 45 point series in Fig 1. For *m* = 2, considering template vector *u*(10) = [*x*(10), *x*(11)], the tolerance regions (shown as green bands in Fig 1) with width 2r can be drawn around each element of vector *u*(10) [e.g. x(10) and x(11)]. For any vector *u*(*j*) = [*x*(*j*), *x*(*j* + 1)], when *x*(*j*) and *x*(*j* + 1) falls into corresponding tolerance regions, then vector *u*(*j*) is counted. As shown in Fig 1, there are two vectors *u*(20) and *u*(35) fulfilling the requirement, e.g. $A_{10}^{2}\left(r\right)=\frac{1}{21}$, Then, we can get *A*^2^(*r*) by means of ergodic *u*(*j*). With the same approach, we can get *B*^2^(*r*). Finally, SampEn can be derived from Eq 6.

SampEn is precisely the negative natural logarithm of an estimate of the conditional probability (CP) that runs of patterns that close (within *r*) for *m* contiguous observations remain close (within the same tolerance width *r*) on next incremental comparisons, and is a useful tool to investigate the dynamics of time series. SampEn assigns a nonnegative number to a time series, with larger values corresponding to greater apparent serial randomness or irregularity and smaller values corresponding to more recognizable features in the data sequence. It is also worthwhile to point out, mathematically similar to ApEn, that the two input parameters, *m* and *r*, must be specified to compute SampEn.

### Optimal Selection of *m* and *r*

Mathematically similar to ApEn, SampEn is a family of parameters and statistics for quantifying system regularity and complexity [26]. Two input parameters, *m* and *r*, must be specified to compute SampEn value, that is, for a specified system, SampEn comparisons must have fixed *m* and *r*, due to variations of the significant dependence on different *m* and *r*.

Thus, we first must determine parameters *m* and *r* values that can capture essential feature of the fMRI dataset structure, and keep the statistical bias of SampEn at an acceptable level. We define *B* to be the number of matches of length *m*, and *A* to be the number of matches of length *m* + 1, so *CP* = *A*/*B*, SampEn(*m*, *r*, *N*) can be expressed as −*ln*(*A*/*B*). The variance of CP can be estimated as [18]:

$$\begin{array}{c} \sigma_{CP}^{2}=\frac{CP(1-CP)}{B}+\frac{1}{B^{2}}\left[K_{A}-K_{B}{\left(CP\right)}^{2}\right] \end{array}$$ (7)

where *K*_*A*_ and *K*_*B*_ are the number of pairs of matching templates of length *m* + 1 and *m* that overlap, respectively. Thus, the standard deviation of SampEn can be estimated by *σ*_*CP*_/*CP*.

Recently, it has been noticed that ApEn(*m*, *r*, *N*) appears to have asymptotic chi-square distribution for large sets of uniformly distributed discrete data [27], and several additional works have pointed out that ApEn(*m*, *r*, *N*) appears asymptotically normal for several simulated weak-dependence processes and electroencephalogram (EEG) time series [28, 29]. However, analytic proofs, even numerical computation, of the distribution of SampEn(*m*, *r*, *N*) are very difficult to achieve due to the very small number of data points for fMRI time series. Following the spirit of a previous study [18], we consider SampEn as an approximately normal distribution, and thus the 95% confidence intervals (CI) for SampEn calculation can be defined as −*log*(*CP*) ± 1.96(*σ*_*CP*_/*CP*), that is, *P*(−1.96*σ*_*CP*_ ≤ *ξ*(*CP*) ≤ 1.96*σ*_*CP*_) = 0.95.

**Fig 2:**
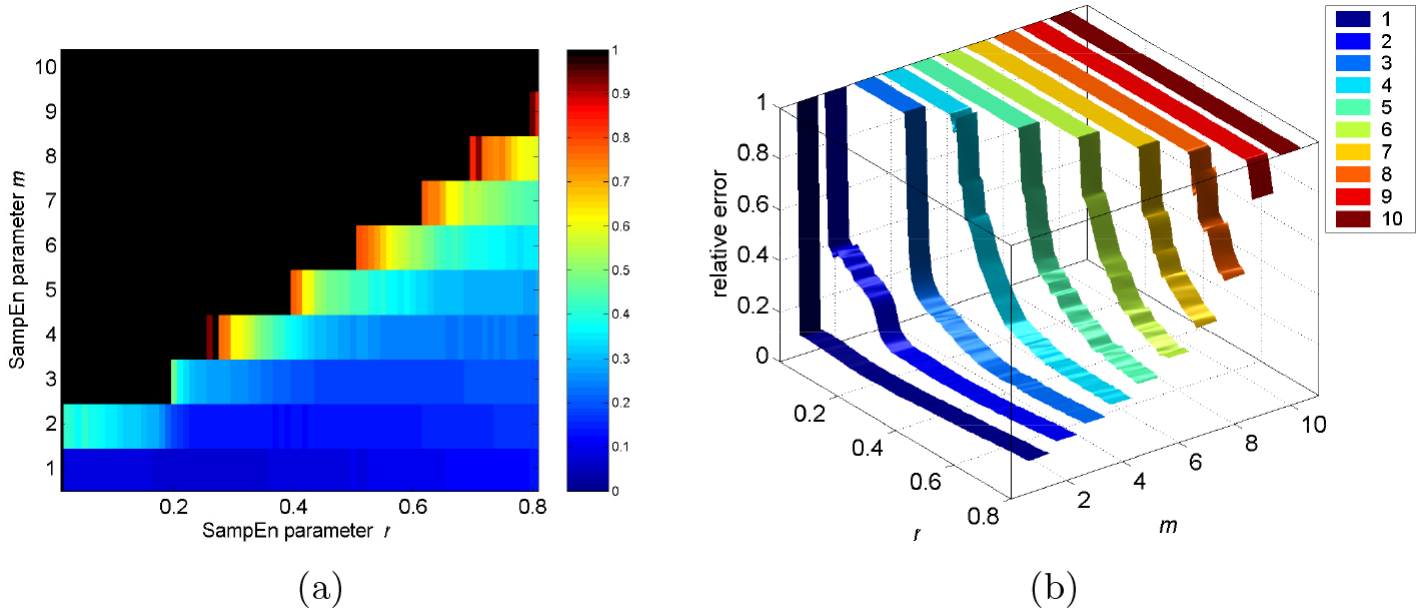
A visual guide to optimal selection of window length (*m*) and tolerance (*r*) parameters for SampEn estimation of fMRI time series of length 128. (a) the median value of relative error of SampEn is shown in pseudocolor. (b) their changes with *m* and *r* is shown as a color ribbon map.

Fig 2 shows a color map of the median value of relative error of SampEn for 900 randomly selected intracerebral voxels, with records of length 128, from 9 subjects in resting state. The relative error of SampEn ranges from 0, a deep blue, to 1, a deep red, and is black where no matches of length *m* are found. Furthermore, based on some theoretical analysis and clinical application, it has been suggested that for *m* = 1 and 2 [30–32], values of *r* between 0.1 to 0.25 SD of the *u*(*i*) data can produce good statistical validity and system identification [33]. Following this criteria, we have selected *m* and *r* to minimize the estimated relative error. Thus, we selected *m* = 1 and *r* = 0.20 to analyze the fMRI dataset, in which the median value of relative error of SampEn for all selected data point is 0.0744, and the 95% CI of the estimate is ∼15% of its value.

Once the optimal *m* and *r* are selected, we quantify system complexity for each intracerebral voxel using the SampEn measure.

### The Wilcoxon Signed Rank Test

The traditional parametric statistics are usually restricted to the assumption that the underlying populations are normally distributed. Since the distribution of SampEn(*m*, *r*, *N*) remains unclear on real fMRI processes [14], we have applied nonparametric statistical strategy instead [34]. Requiring only minimal assumptions for validity, nonparametric tests provides a flexible and intuitive paradigm for the statistical analysis of data from functional neuroimaging experiments [35]. In particular, we are interested in searching over the whole brain for significant shift in location due to the application of the different experimental paradigms, and we have used the Wilcoxon signed rank test [36].

Suppose that we have obtained in total 2*n* observations *X*_1_, ⋅⋅⋅, *X*_*n*_ (neutral-blank) and *Y*_1_, ⋅⋅⋅, *Y*_*n*_ (threat-neutral) for *n* subjects in two conditions. Let *Z*_*i*_ = *Y*_*i*_ − *X*_*i*_ and take as our model

$$\begin{array}{c} Z_{i}=\theta +e_{i},\;\;\;\;\;i=1,\cdot \cdot \cdot ,n, \end{array}$$ (8)

where the *e*_*i*_ is unobservable random variables and mutually independent, and *θ* is the unknown *treatment* effect. To test the hypothesis

$$\begin{array}{c} H_{0}:\theta =0, \end{array}$$ (9)

form the absolute difference |*Z*_1_|, ⋅⋅⋅, |*Z*_*n*_|. Let *R*_*i*_ denote the rank of |*Z*_*i*_| in the joint ranking from least to greatest of |*Z*_1_|, ⋅⋅⋅, |*Z*_*n*_|. Forming the *n* products *R*_1_ *ψ*_1_, ⋅⋅⋅, *R*_*n*_ *ψ*_*n*_, where *ψ*_*i*_, *i* = 1, ⋅⋅⋅, *n*, defined as indicator variables, is Heaviside function, and set

$$\begin{array}{c} T^{+}=\sum_{i=1}^{n}R_{i}\psi_{i}. \end{array}$$ (10)

The product *R*_*i*_ *ψ*_*i*_ is known as the positive signed rank of *Z*_*i*_. It takes on the value zero if *Z*_*i*_ is negative and is equal to the rank of |*Z*_*i*_| when *Z*_*i*_ is positive. The statistic *T*^+^ is the sum of the positive signed ranks.

Suppose that the test is made at the 4% level of significance, that is, *α* = 0.04. A two-sided test of $H_{0}$ versus the alternative *θ* ≠ 0 is,

$$\begin{array}{c} \begin{array}{cccc} & \text{reject}\;\;H_{0} & \;\text{if}\; & T^{+}\geq t\left(\alpha_{2},n\right)\;\;\text{or}\;\;T^{+}\leq \frac{n(n+1)}{2}-t\left(\alpha_{1},n\right),\\ & \text{accept}\;\;H_{0} & \;\text{if}\; & \frac{n(n+1)}{2}-t\left(\alpha_{1},n\right)<T^{+}<t\left(\alpha_{2},n\right), \end{array} \end{array}$$ (11)

where *α* = *α*_1_ + *α*_2_ = 0.02 + 0.02 = 0.04.

Here, the critical value at the *α* = 0.04 level is *t*(0.02, 9) = 40 for *θ* > 0 and $\frac{9(10)}{2}-t\left(0.02,9\right)=45-40=5$ for *θ* < 0.

## Results and Discussion

**Fig 3:**
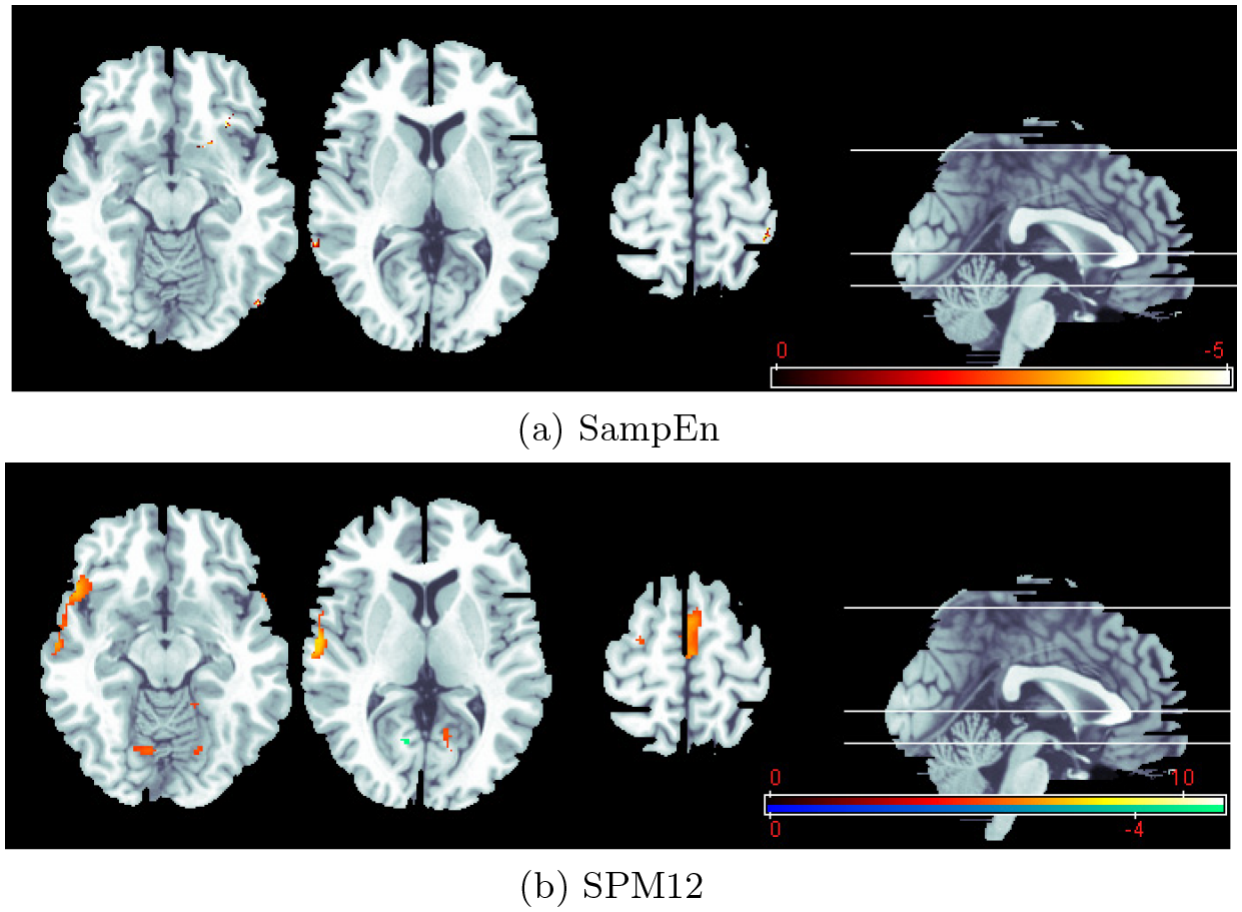
The activated clusters detected by (a) SampEn, in which statistical *T*^+^ map reveals an activation change in two experimental paradigms (neutral-blank to threat-neutral); (b) SPM12, in which statistical *T* map reveals activations during processing of neutral-blank (hot orange) and threat-neutral (winter blue).

In Fig 3(a), we present a threshold statistic *T*^+^ map (*p* < 0.04) from complexity analysis during processing of neutral-blank and threat-neutral. For comparative purposes, Fig 3(b) also shows the detection results from SPM12 during processing of neutral-blank (hot orange) and threat-neutral (winter blue) on the same slices. As expected, one can find that the complexity-based result is very different from that from the most commonly used SPM12. Two factor may contribute these differences. First, conventional SPM method mainly evaluates the regional hemodynamic changes in response to different task activation (listening neutral word to blank, or listening threat-related word to listening neutral word) in a single experiment, while SampEn method detects brain complexity changes in two experiments of different conditions. Furthermore, SPM is a model-driven method, which requires that the temporal dynamics of the activation response be consistent to *a priori* expectant hemodynamic response, while the data-driven method SampEn evaluates just the complexity of specific sequential fMRI data.

In addition, it should also be emphasized that the activation region overall comes from *θ* < 0, that is, there are higher SampEn values in neutral-blank condition than in threat-neutral. In fact, even at 25% level of significance, a rather loose significance level, we cannot find the *θ* > 0 activation. SampEn is a regularity statistic that quantifies the unpredictability of fluctuations in a time series. A larger SampEn value corresponds to greater apparent process randomness or serial irregularity, and a smaller value corresponds to more instances of recognizable features or patterns in the data. To some extent, we can take the brain as a input/output system in which the output signal is the result of the convolution of the input signal with an impulse response (hemodynamic response). The time course of the block design is a square-wave, and neutral-blank paradigm has a higher pink-to-pink value than threat-neutral. Thus, the neutral-blank block design produces higher predictability than threat-neutral. But, it is somewhat puzzling that these predictable changes related to the experimental paradigm take place at the whole brain. We believe that changed SampEn values imply changed hemodynamic response invoked by experimentally controlled stimuli, but the character of SampEn needs to be further investigated to substantiate such claim.

In conclusion, this study presents a complexity approach based on SampEn analysis. It could be considered as a valuable complementary method to present classical fMRI analysis, and it could help improving the understanding of human brain functions.
